# Use of Indicator Kriging to Investigate Schistosomiasis in Minas Gerais State, Brazil

**DOI:** 10.1155/2012/837428

**Published:** 2012-01-12

**Authors:** Ricardo J. P. S. Guimarães, Corina C. Freitas, Luciano V. Dutra, Carlos A. Felgueiras, Sandra C. Drummond, Sandra H. C. Tibiriçá, Guilherme Oliveira, Omar S. Carvalho

**Affiliations:** ^1^Divisão de Processamento de Imagens (DPI), Instituto Nacional de Pesquisas Espaciais (INPE), Avenida dos Astronautas, 1758, Jd. Granja, 12227-010 São José dos Campos, SP, Brazil; ^2^Laboratório de Geoprocessamento (LabGeo)/Instituto Evandro Chagas (IEC), Rodovia BR316, km7, s/n, Levilândia, 67030-000 Ananindeua, PA, Brazil; ^3^Serviço de Saúde Ambiental/Fundação Nacional de Saúde (FUNASA), Belo Horizonte, Rua Espírito Santo, 500, Centro, 30160-030 Belo Horizonte, MG, Brazil; ^4^Departamento de Clínica Médica/Faculdade de Medicina/Universidade Federal de Juiz de Fora (UFJF), Rua José Lourenço Kelmer, s/n, Bairro São Pedro, 36036-900 Juiz de Fora, MG, Brazil; ^5^Laboratório de Parasitologia Celular e Molecular (LPCM)/Centro de Pesquisas René Rachou (CPqRR)/Fiocruz-MG, Avenida Augusto de Lima, 1715, Barro Preto, 30190-002 Belo Horizonte, MG, Brazil; ^6^Laboratório de Helmintologia e Malacologia Médica (LHMM)-Centro de Pesquisas René Rachou (CPqRR)/Fiocruz-MG, Avenida Augusto de Lima, 1715, Barro Preto, 30190-002 Belo Horizonte, MG, Brazil

## Abstract

Geographic Information Systems (GISs) are composed of useful tools to map and to model the spatial distribution of events that have geographic importance as schistosomiasis. This paper is a review of the use the indicator kriging, implemented on the Georeferenced Information Processing System (SPRING) to make inferences about the prevalence of schistosomiasis and the presence of the species of *Biomphalaria*, intermediate hosts of *Schistosoma mansoni*, in areas without this information, in the Minas Gerais State, Brazil. The results were two maps. The first one was a map of *Biomphalaria* species, and the second was a new map of estimated prevalence of schistosomiasis. The obtained results showed that the indicator kriging can be used to better allocate resources for study and control of schistosomiasis in areas with transmission or the possibility of disease transmission.

## 1. Introduction

Schistosomiasis mansoni is an endemic disease, typical of developing countries [1, 2]. In Brazil, the schistosomiasis is caused by the etiological agent *Schistosoma mansoni*, whose intermediate host is species of mollusk of the *Biomphalaria* genus.

The *S. mansoni* was introduced in Brazil by the African slavery trade during the sixteenth century [3]. The Brazilian port of Salvador and Recife received most of the slaves [4], originated from endemic regions. In the early eighteenth century, there was a large migration of slave labor due to the decline of sugar production in the Northeast of Brazil and the discovery of gold and diamond in the Minas Gerais State. It is estimated that one fifth of the population at that time moved to Minas Gerais [5], using the “ways of São Francisco” [6] as the main access route. Probably, in these early migrants also came to schistosomiasis.

In Minas Gerais, there are seven species of *Biomphalaria* genus: *B. glabrata*, *B. straminea*, *B. tenagophila*, *B. peregrina*, *B. schrami*, *B. intermedia*, and *B. occidentalis* [7–9]. Come in these, only *B. glabrata*, *B. tenagophila*, and *B. straminea* have been found naturally infected by *S. mansoni* [10, 11]. *B. glabrata* is of great epidemiologic importance, due to its extensive geographic distribution, high infection indices, and effectiveness in the schistosomiasis transmission. Moreover, its distribution is almost always associated with disease occurrence [12]. *B. tenagophila *was found naturally infected by* S. mansoni *in state of Minas Gerais, and it is responsible for the focus maintenance in the city of Itajubá [13]. *B. straminea*, although had not been found infected in state of Minas Gerais, was considered responsible for Paracatu's focus [14].

They are commonly found in a wide of habitats, both natural (streams, creeks, ponds, swamps) and artificial (irrigation ditches, small dams), particularly in shallow and slow running waters (less than 30 cm/s), where the substratum can be the muddy or rocky bed and with floating or rooted vegetation, pH between 6 and 8, NaCl content below 3 by 1000, and mean temperature between 20 and 25 degrees C [15–17].

The study of the habitat of these mollusks, as well as their behavior in relation to the climate, results in valuable information when the goal is the disease transmission control [18].

Environmental and socioeconomic factors may influence the spatial distribution of schistosomiasis. Under these circumstances, the Geographic Information System (GIS) can be applied to characterize, to better understand the interconnection of these factors, and to provide a more complete picture of disease transmission [19]. GIS allows a complex analysis of a large number of information and displays the results of this analysis in graphical maps. These techniques have become important tools for the design and implementation of control programs [20], enabling a better distribution of state resources to allow a direction more suitable for disease control [21–23]. Among these tools, we can cite the indicator kriging, which allows to data spatialization aiming at map generation. It also gives information about inference uncertainties that can be used as quality restrictions of the classification process.

This study is a review of the use the indicator kriging of the Georeferenced Information Processing System (SPRING) to make inferences about the presence of the species of *Biomphalaria* (*B. glabrata*, *B. tenagophila*,* and/or B. straminea*), intermediate hosts of *Schistosoma mansoni*. Also, using numerical indicator kriging, a new map of estimated prevalence of schistosomiasis, in areas without information in the Minas Gerais State, Brazil, is presented.

## 2. Methodology

Kriging may be defined as a technique of statistical inference, which allows the estimation of values and the uncertainties associated with the attribute during the spatialization of a sample property [24].

To achieve the objectives, two approaches have been considered: categorical and numerical indicator kriging. The categorical indicator kriging was based on the information of the mollusks species, and the numerical indicator kriging used data from the prevalence of schistosomiasis.

The procedure for adjustment of the semivariogram is not straightforward and automatic, but interactive, because the interpreter does the first adjustment and checks the adequacy of the theoretical model [25]. After the models fitted for each class (categorical) or quartile (numerical), the indicator kriging was applied to obtain an approximation of the conditional distribution function of random variables.

The numerical indicator kriging was conducted in the entire state using the schistosomiasis prevalence data (lower quantile, median and upper quantile) from 999 localities.

The categorical indicator kriging was performed in each of the fifteen river basins (Buranhém, Doce, Grande, Itabapoana, Itanhém, Itapemirim, Jequitinhonha, Jucuruçu, Mucuri, Paraíba do Sul, Paranaíba, Pardo, Piracicaba/Jaguari, São Francisco and, São Mateus) using the mollusk data. The mollusk attributes (class of species and localization) were distributed along the drainage network of 15 River Basins, according to the methodology used by Guimarães et al. [22]. The classes used for this study were defined as *B. glabrata*, *B. tenagophila*, *B. straminea*, *B. glabrata + B. tenagophila*, *B. glabrata + B. straminea*, *B. tenagophila + B. straminea*, *B. glabrata + B. tenagophila + B. straminea*, and without *Biomphalaria*. The class without *Biomphalaria* includes information about the nonoccurrence of *Biomphalaria* species or information about non-transmitter species in Brazil, such as *B. peregrina*,* B. schrammi*, *B. intermedia*, and *B. occidentalis*.

The indicator kriging was done in the software SPRING [26]. In Appendix A is described the geostatistical modeling used in the indicator kriging.

### 2.1. Data Set

**Figure 1:**
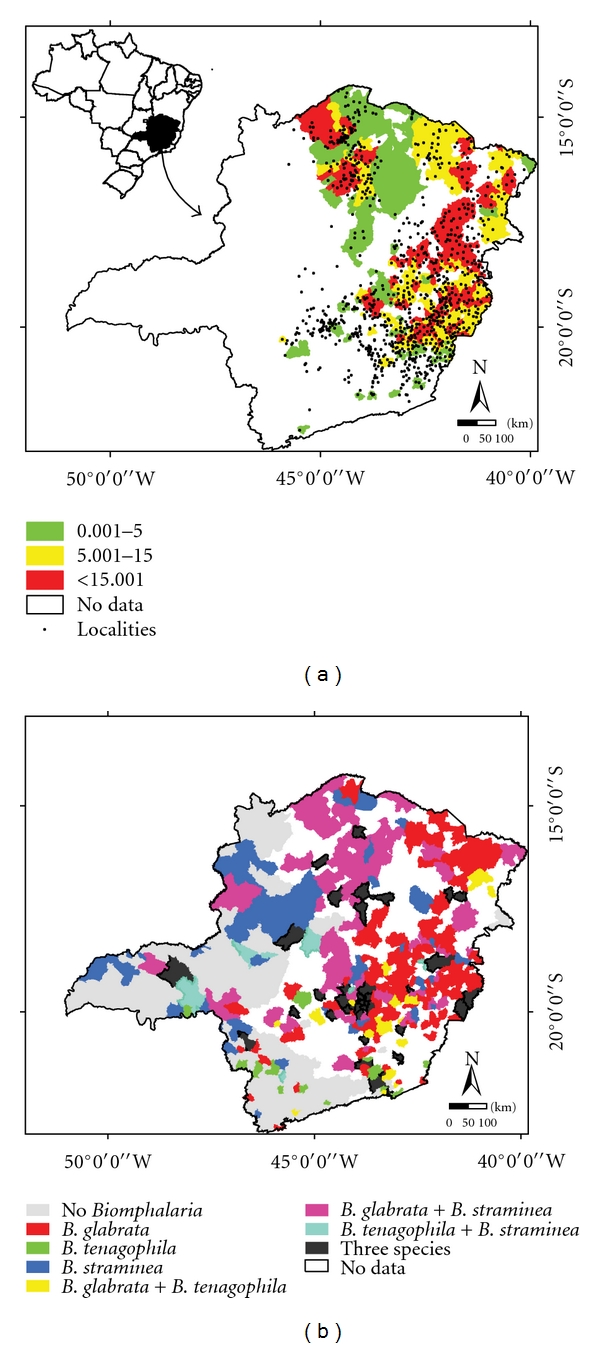
(a) Distribution of the schistosomiasis prevalence on the municipalities (%) 0.001–5.000 (green), 5.001–15.000 (yellow), and above 15.001 (red), and localities (black points); (b) distribution of the *Biomphalaria* species in Minas Gerais.

Schistosomiasis prevalence values (*Pv*) were obtained from the Brazilian Schistosomiasis Control Program (PCE) through the Annual Reports of the Secretary of Public Health Surveillance (SVS) and the Secretary of Health in the State of Minas Gerais (SESMG). The PCE in Minas Gerais had its beginning in 1986, and since 2000 has been under the coordination of the SESMG in collaboration with Municipal Health Systems. The PCE prevalence information is available for municipalities and localities [18]. The Kato Katz technique is the methodology used to determine prevalence, examining one slide per person. The spatial distribution of the schistosomiasis prevalence is presented in Figure 1(a), for the 255 municipalities and for the 999 localities used in this study.

Data on the distribution of *Biomphalaria* mollusks were provided by the Laboratory of Helminthiasis and Medical Malacology of the René Rachou Research Center (CPqRR/Fiocruz-MG). Mollusks were collected in breeding places from different municipalities in Minas Gerais at different periods, using scoops and tweezers, and then packed to be transported to the laboratory [27]. Specific identification was performed according to the morphology of the shells, reproductive system, and renal ridge of the mollusks [28–33], and also by low stringency polymerase chain reaction and restriction fragment length polymorphism [34]. The spatial distribution of the *Biomphalaria* species data are presented in Figure 1(b). 

### 2.2. Test of the Hypothesis for Differences between PCE and Indicator Kriging Estimated Prevalences

To assess whether the estimates made by kriging methods were close to those obtained by PCE, initially estimates were made by municipalities, by averaging the estimated prevalence for all the grid points belonging to that municipality.

A regression line was then adjusted, using the prevalence provided by the PCE as dependent variable and the prevalence provided by kriging as independent variable, that is,

(1) $$P_{\text{PCE}}=\beta_{0}+\beta_{1}P_{K},$$

where *P*_PCE_ and *P*_*K*_ are the PCE and kriging prevalences, respectively.

A hypothesis test was then performed to determine, with a 95% confidence level, whether the intercept was zero and the slope parameter was equal to 1:

(2) $$\begin{array}{c} H_{0}:\beta_{0}=0,\;\;\beta_{1}=1,\\ H_{1}:\beta_{0}\neq 0\;\text{or}\;\beta_{1}\neq 1. \end{array}$$

If the null hypothesis is accepted, it can be concluded that, in average, the kriging estimates are equal to PCE prevalences at a 95% confidence level.

## 3. Results and Discussions

The indicator kriging procedure, based on the fitted semivariograms, was applied using the sample data presented in Figure 1, to generate a regular grid of 250 meters of resolution (*x*, *y*) over the Minas Gerais State.

The following results were obtained to achieve the objectives.

### 3.1. Using Categorical Data (Thematic)

**Figure 2:**
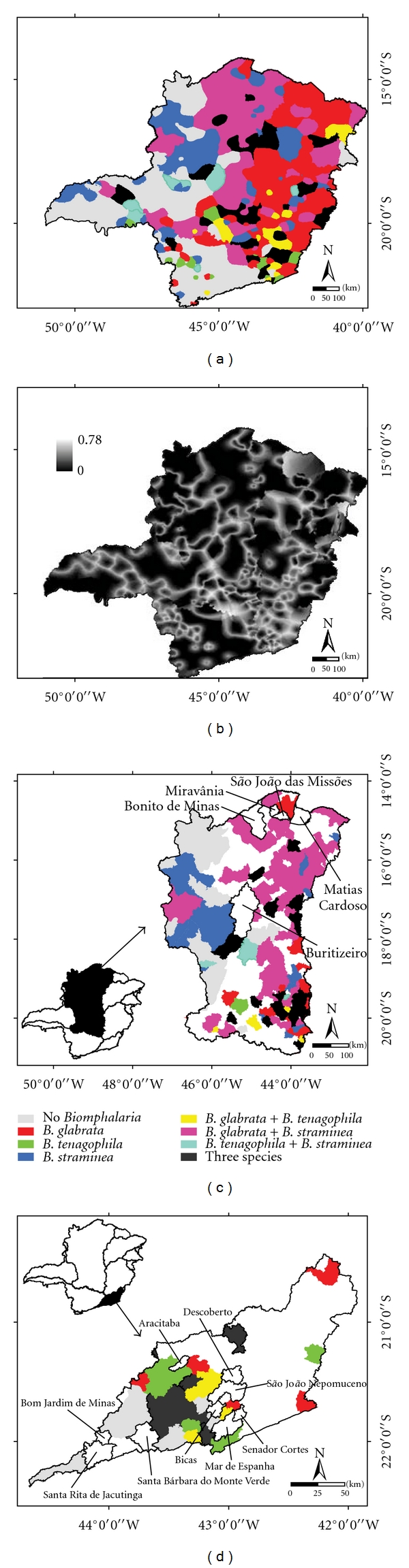
(a) Map of the estimated species distribution, (b) map of the uncertainties, (c) historical *Biomphalaria* species in São Francisco River Basin, and in (d) Paraíba do Sul River Basin. (Source: [18, 22]).

The resulting map of the species distribution generated by applying the mode estimator (Appendix A.2.—(A.6)) is presented in Figure 2(a).

Figure 2(b) presents a map of the uncertainties associated with the classification, computed by using (A.7) of Appendix A.2. The map of uncertainties shows that the higher uncertainties are concentrated among class transition areas.

The methodology was validated using a sampling procedure. The fieldwork was conducted in the São Francisco and Paraíba do Sul River Basins where no information existed about the presence of the mollusks. More details about the results for the São Francisco and Paraíba do Sul River Basins can be found in Guimarães et al. [22] and Guimarães [18] and Tibiriçá et al. [9].

The research of mollusks was accomplished in five municipalities in the São Francisco River Basin (SFRB) and in nine municipalities in the Paraíba do Sul River Basin (PSRB). The mollusks collected were sent to the analysis of the species in the Laboratory of Helminthiasis and Medical Malacology of the René Rachou Research Center (CPqRR/Fiocruz-MG). Also, the mollusks collected in the PSRB were identified at the Parasitology Laboratory in the Federal University of Juiz de Fora (UFJF) and the Entomology Laboratory of the GRS/JF, Secretary of Health in the State of Minas Gerais (SESMG). Collection and identification of the mollusks were performed according to the methodology described in Section 2.1.

Figures 2(c) and 2(d) show the historical *Biomphalaria* species (Figure 1(b)) at the surveyed municipalities of São Francisco and Paraíba do Sul River Basins, respectively.

Table 1 presents the estimated and found species, as well as the value of the uncertainty mean for searched municipality, and the collection points, where these species had been found.

To explain the differences in the two basins, some considerations should be made.  Figure 2 shows the spatial distribution of *Biomphalaria* species according to historical data. We can observe from this figure that the SFRB (Figure 2(c)) has a better spatial distribution of species surveyed than the PSRB (Figure 2(d)). 

The municipalities surveyed in the SFRB had 100% accuracy with at least one specie estimated, but this value for the PSRB was 66.67%.

About 50% of the municipalities of SFRB have historical information about the *Biomphalaria* species in one of eight classes (*B. glabrata*, *B. tenagophila*, *B. straminea*, *B. glabrata *+ *B. tenagophila*, *B. glabrata *+ *B. straminea*, *B. tenagophila *+ *B. straminea*, *B. glabrata *+ *B. tenagophila *+ *B. straminea, *and without *Biomphalaria*). However, for PSRB, this information is only 32% of the municipalities and one of five classes (*B. glabrata*, *B. tenagophila*, *B. glabrata *+ *B. tenagophila*, *B. glabrata *+ *B. tenagophila *+ *B. straminea,* and without *Biomphalaria*).

As kriging is affected by the amount and spatial distribution of input data, this may explain the differences in the two basins.

This fact is also reflected in the uncertainties of the estimates. Comparing the values presented in Table 1, the SFRB had an overall uncertainty mean of 0.232, and the PSRB had an overall uncertainty mean of 0.332. Therefore, the overall uncertainty for PSRB is 43.1% greater than for SFRB.

### 3.2. Using Numerical Data

**Figure 3:**
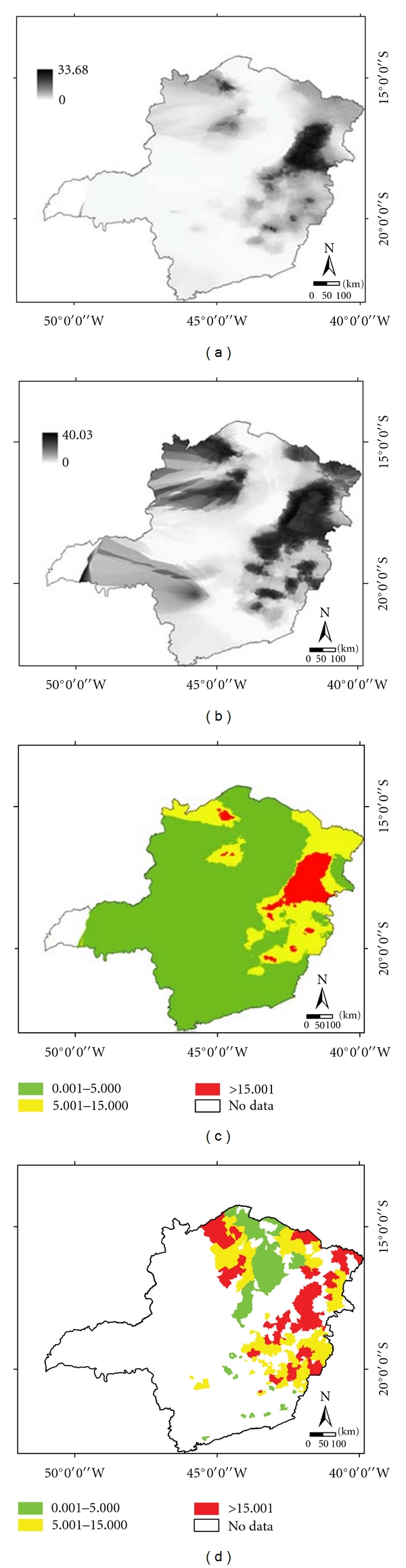
(a) Estimated prevalence by kriging, (b) map of the uncertainties, (c) estimated prevalence by kriging by type of classes, (d) mean estimated prevalence by kriging for 255 municipalities.

Figure 3 shows the spatial distribution of schistosomiasis-estimated prevalence by kriging (Figure 3(a)), map of the uncertainties (Figure 3(b)), estimated prevalence by kriging by type of classes (Figure 3(c)), and in Figure 3(d) the mean estimated prevalence by kriging for the 255 municipalities where the PCE prevalence information is available.

**Figure 4:**
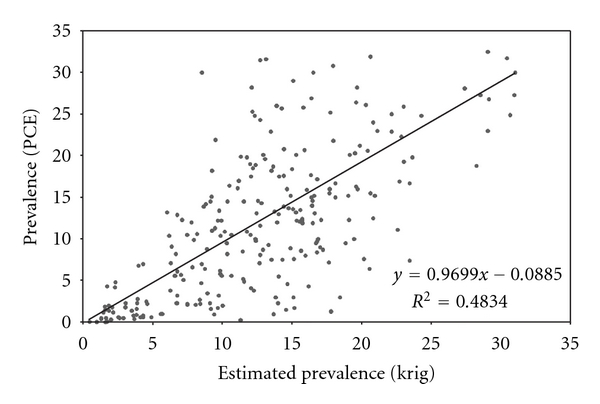
PCE- and kriging-estimated prevalences for 255 municipalities.

The PCE prevalence values (Figure 1(a)) and the respective kriging estimates (Figure 3(d)) were plotted together.  Figure 4 shows the scatter plot as well as the regression line. The hypothesis test (A.12) was performed, and the null hypothesis was accepted, indicating that there is no significant difference between the PCE prevalence and the kriging prevalence means, with a significance level of 0.05.

Table 2 presents the comparison between the prevalence of PCE and the prevalence estimated by kriging by type of classes: low (prevalence among 0.001 to 5), medium (5.001 to 15), and high prevalence (above 15).

From Table 2, it can be noted that

- 60.8% of the municipalities are estimated in the same class as they belong;
- 37.2% of the municipalities had the prevalence estimated in the adjacent class, that is, from low to medium class, from medium to high class, from high to medium class, or from medium to high class;
- less than 2% of the municipalities had the low prevalence estimated a high class;
- when the estimated class is not in the same class of the PCE, the kriging has a trend of about 25.9% to overestimate and about 13.3% to underestimate the prevalence values.

## 4. Conclusions and Future Work

Indicator kriging showed to be a rather robust tool since its results presented a very good agreement with the field findings. This tool allowed to determine and to delimit, respectively, the distribution of the *Biomphalaria* species and the areas of risk (map of uncertainty of the *Biomphalaria* species).

Kriging is an auxiliary useful tool to guide the fieldwork, indicating the places with higher probability of occurrence of the considered species, with particular attention to those species that are more important for disease transmission. The results of this tool can be used to better allocate the always limited resources for distribution studies and the development of strategies for mollusk control.

Some important issues, related to the nature and precision of the *Biomphalaria* species data, need to be considered when looking at the results: the data were obtained from historical records (most occurring before the broad usage of GPS equipment), and the information is given in a municipality level basis. Because of this, an assumption was made that the species found in the municipalities are uniformly distributed inside the municipality drainage network. The authors believe, however, that other type of distribution hypothesis would not greatly affect the results. 

To improve the accuracy of an estimate using kriging, it would be necessary to obtain data with better location and spatial distribution of the information collected in the fieldwork.

Also, the kriging proved to be a suitable tool, and their results showed a good agreement with the PCE data.

This technique can be used to estimate the schistosomiasis prevalence in the municipalities of Minas Gerais where the prevalence is not determined by the PCE. The results of this tool can be used to better allocate resources for studies in areas with medium and high prevalence.

The entire methodology of this study used free software allowing the playback of the methodology in other states of Brazil where there is no information about the type of *Biomphalaria* and/or schistosomiasis prevalence at no cost.

Conditioned to appropriate funds existence, an extensive malacological survey is recommended for better evaluation of the methodology and also GPS utilization in all future fieldworks.

It is also recommended to obtain data on the schistosomiasis prevalence in western Minas Gerais (nonendemic region). Thus, one can obtain a better estimate of prevalence at the state level and not only in the endemic area.

## Figures and Tables

**Table 1:** List of surveyed municipalities and their attributes found in the San Francisco and Paraíba do Sul River Basins (Source: [9, 18, 22]).

River basin	Municipalities	Estimated species	Uncertainty mean	Found species
	Bonito de Minas	*B. straminea,* *B. glabrata*	0.002	*B. straminea,* *B. glabrata*
	Buritizeiro	*B. straminea,* *B. glabrata*	0.250	*B. straminea*
São Francisco	Matias Cardoso	*B. straminea,* *B. glabrata*	0.290	*B. straminea*
	Miravânia	*B. straminea,* *B. glabrata*	0.358	*B. straminea*
	São João das Missões	*B. straminea,* *B. glabrata*	0.279	*B. straminea*

	Aracitaba	*B. glabrata,* *B. tenagophila*	0.281	*Other* ^ a^ * (Bp)*
	Bicas	*B. glabrata,* *B. tenagophila*	0.419	*B. glabrata,* *B. tenagophila*
	Bom Jardim de Minas	Without *Biomphalaria *	0	Without *Biomphalaria *
	Descoberto	*B. glabrata,* *B. tenagophila,* *B. straminea*	0.487	*B. glabrata,* *B. tenagophila*
Paraíba do Sul	Mar de Espanha	*B. glabrata,* *B. tenagophila,* *B. straminea*	0.525	*B. tenagophila, Other (Bp,Bo)*
	Santa Bárbara do Monte Verde	*B. glabrata,* *B. tenagophila,* *B. straminea*	0.302	*Other (Bp)*
	Santa Rita de Jacutinga	Without *Biomphalaria *	0	*B. tenagophila, Other (Bp)*
	São João Nepomuceno	*B. glabrata,* *B. tenagophila*	0.438	*B. glabrata,* *B. tenagophila,* *Other (Bp)*
	Senador Cortes	*B. glabrata,* *B. tenagophila*	0.535	*Other (Bp)*

^a^Other: Class without *Biomphalaria*, species found in this class is not the transmitter of schistosomiasis; Bp: *B. peregrina*; Bo:* B. occidentalis.*

**Table 2:** Comparison between PCE and kriging prevalences for the 255 municipalities.

		PCE			
		Low	Medium	High	Total
Kriging	Low	37	2	0	39
	Medium	28	62	32	122
	High	5	33	56	94

	Total	70	97	88	255

## Appendix A

### A.

#### A.1. Indicator Kriging

A spatial attribute inside a region *A* of the earth surface can be modeled, from a geostatistical point of view, as a random function [35, 36]. For each spatial position **u** ∈ *A*, the attribute value of a spatial data is modeled as a random variable (RV) *Z*(**u**), which can assume different values with an associated probability of occurrence. In the *n* sample positions **u**_*α*_, *α* = 1,2,…, *n*, the values *Z*(**u**) are considered deterministic, that is, they can be considered RV's with probability 100% of occurrence. The distribution function of *Z*(**u**), conditioned to *n* sample points, can be defined by Deutsch and Journel [36]

(A.1) $$\begin{array}{c} F\left(u;z\;|\;\left(n\right)\right)=\text{Prob}\left\{Z\left(u\right)\leq z\;|\;\left(n\right)\right\}\\ \;\;\;\;\;\text{for}\;\text{a}\;\text{numerical}\;\text{attribute},\\ F\left(u;z\;|\;\left(n\right)\right)=\text{Prob}\left\{Z\left(u\right)=z\;|\;\left(n\right)\right\}\\ \;\;\;\;\;\text{for}\;\text{a}\;\text{categorical}\;\text{attribute}. \end{array}$$

The function *F*(**u**; *z* | (*n*)) models the uncertainty about the values of *z*(**u**), in nonsampled positions **u**, conditioned to the *n* samples. This function can be evaluated from the inference procedure-denominated indicator kriging.

The indicator codification of the RV *Z*(**u**), for a cutoff value *z* = *z*_*k*_, generates the indicator RV  *I*(**u**; *z*_*k*_) using the following nonlinear mapping function:

(A.2) $$\begin{array}{c} I\left(u;z_{k}\right)=\{\begin{array}{c} 1\;\text{if}\;Z\left(u\right)\leq z_{k}\\ 0\;\text{if}\;Z\left(u\right)>z_{k} \end{array}\text{for}\;\text{a}\;\text{numerical}\;\text{attribute},\\ I\left(u;z_{k}\right)=\{\begin{array}{c} 1\;\text{if}\;Z\left(u\right)=z_{k}\\ 0\;\text{if}\;Z\left(u\right)\neq z_{k} \end{array}\text{for}\;\text{a}\;\text{categorical}\;\text{attribute}. \end{array}$$

The cutoff values, *z*_*k*_, *k* = 1,2 …, *K*, are defined depending on the number of samples. It is necessary that the amount of codified samples with value 1 are enough for defining, with success, a variography model for each cutoff value [37].

The conditional expectation of the indicator RV  *I*(**u**; *z*_*k*_) is computed by

(A.3) $$\begin{array}{cc} E\left\{I\left(\left(u;z_{k}\right)\;|\;\left(n\right)\right)\right\} & =1\cdot \text{Prob}\left\{I\left(u;z_{k}\right)=1\;|\;\left(n\right)\right\}\\ & \;+0\cdot \text{Prob}\left\{I\left(u;z_{k}\right)=0\;|\;\left(n\right)\right\}\\ & =1\cdot \text{Prob}\left\{I\left(u;z_{k}\right)=1\;|\;\left(n\right)\right\}\\ & =F^{*}\left(u;z_{k}\;|\;\left(n\right)\right). \end{array}$$

This estimation, made through ordinary kriging over the indicator values, yields a least-square estimate for the distribution function at the cutoff value *z*_*k*_ [36]. A set of *F**(**u**; *z*_*k*_ | (*n*)) estimates, obtained for different *k* cutoff values, can lead to an approximation of the conditional distribution function (cdf) of *Z*(**u**).

The expression of the ordinary indicator kriging estimator is given by:

(A.4) $$F_{O}^{*}\left(u;z_{k}\;|\;\left(n\right)\right)=\overset{n(u)}{\underset{\alpha =1}{\sum }}\lambda_{O\alpha }\left(u;z_{k}\right)i\left(u_{\alpha };z_{k}\right).$$

The weights *λ*_*Oα*_(**u**; *z*_*k*_) are obtained by solving the following system of equations:

(A.5) $$\begin{array}{c} \overset{n(u)}{\underset{\beta =1}{\sum }}\lambda_{O\beta }\left(u;z_{k}\right)C_{1}\left(h_{\alpha \beta };z_{k}\right)+\phi \left(u;z_{k}\right)=C_{1}\left(h_{\alpha };z_{k}\right)\\ \;\;\;\;\;\;\;\;\;\;\;\;\forall \alpha =1,2,\ldots ,n\left(u\right),\\ \overset{n(u)}{\underset{\beta =1}{\sum }}\lambda_{O\beta }\left(u;z_{k}\right)=1, \end{array}$$

where *ϕ*(**u**; *z*_*k*_) is a Lagrange parameter, *h*_*αβ*_ is the vector defined by the positions **u**_*α*_  and  **u**_*β*_, *h*_*α*_ is the vector defined by the positions **u**_*α*_  and  **u**, *C*_1_(*h*_*α*_; *z*_*k*_) is the autocovariance defined by *h*_*αβ*_, and  *C*_1_(*h*_*α*_; *z*_*k*_) is the covariance defined by *h*_*α*_. The autocovariance is determined by the theoretical variography model defined by the set *I* when *z* = *z*_*k*_.

The indicator kriging, simple or ordinary, gives, for each cutoff value *k*, an estimator which is also the better least square estimator of the conditional expectation of the RV *I*(**u**; *z*_*k*_). Using this property, it is possible to compute estimates of the cdf values of *Z*(**u**) for several values of *z*_*k*_, belonging to the *Z*(**u**) domain. The set of the estimated values of the cdf's of *Z*(**u**), for the cutoff values, is considered a discrete approximation of the real cdf of *Z*(**u**). The greater the number of cutoff values, the better the approximation.

The indicator kriging is nonparametric. It does not consider any type of a prior distribution for the random variable. Instead, it permits the construction of a discrete approximation of the cdf of *Z*(**u**). The values of the discrete probabilities can be directly used to estimate the values that characterize the distribution, such as average, variance, mode, and quantiles.

#### A.2. Estimation and Uncertainty Evaluation for Categorical (Thematic) Data

The optimal estimation *z**(**u**), for a categorical attribute represented by *K* classes *c*_*k*_, *k* = 1,…, *K*, can be defined as

(A.6) $$z^{*}\left(u\right)=c_{k}\;\text{iff}\;p_{k}\left(u\right)>p_{i}\left(u\right)\;\forall i,k=1,\ldots ,K,\;i\neq k,$$

where *p*_*k*_(**u**) = *F**(**u**; *z*_*k*_ | (*n*)), when *z*_*k*_ = *c*_*k*_, is the estimated probability of the class *k* at the location **u**. This estimator is known as the mode estimator since it considers the highest probability, and the classifier that assigns classes at each position **u** using the mode estimator is known as mode classifier.

The species classification probability for each position, inside the region of interest, is obtained with the values for indication (0 or 1) of the neighboring known samples (nearer) of **u**. This probability is estimated by using ordinary kriging, where the weighted value of each neighboring sample of **u** is calculated considering the variability model defined by the semivariograms for each class.

Usually, inferences about local uncertainties for categorical attributes, Unc(**u**), are made using the complement of the mode probability, which is defined as

(A.7) $$\text{Unc}\left(u\right)=1-\overset{K}{\underset{k=1}{\mathrm{Max}}}\left[p_{k}\left(u\right)\right].$$

The mode estimator, as defined in (A.4), has the advantage of classifying all locations **u** inside the region of interest. An alternate methodology consists in considering the uncertainties as restrictions to the classification process. In this case, only locations with an uncertainty below a predefined threshold (*U*_max⁡_) are classified, that is

(A.8) $$z^{*}\left(u\right)=\{\begin{array}{c} c_{k}\;\text{iff}\left(p_{k}\left(u\right)>p_{i}\left(u\right)\wedge \text{Unc}\left(u\right)<U_{\max}\right)\\ \;\;\;\;\;\;\forall i,k=1,\ldots ,K,\;i\neq k,\\ \phi \;\text{otherwise}, \end{array}$$

where *z**(**u**) = *ϕ* means that the value was not estimated or the location was not classified.

#### A.3. Estimation and Uncertainty Evaluation for Numerical Data

For numerical attributes are estimated univariate cumulative probabilities *p*_*k*_(**u**), *k* = 1,…, *K*, of the cutoff values *k*. These probabilities are used to infer the value and uncertainty in the RV location **u** not sampled. The estimated value of the numerical RV may be the mean or median of the distribution. The uncertainty Unc(**u**) can be determined by the variance *σ* ^2^ = *E*{(*Z*(**u**) − *E*{(*Z*(**u**)})^2^}, and this variance can be used to define confidence intervals of type

(A.9) $$\text{Prob}\left\{Z\left(u\right)\in \left[z^{*}\left(u\right)\pm 2\sigma \left(u\right)\right]\right\}≅0.95$$

when the variable presents a symmetry level that allows to assume the normality hypothesis.

For distributions highly asymmetrical, a more robust measure is the interquartile range defined as the difference between the highest and lowest quartile:

(A.10) $$Q=z_{0.75}^{*}\left(u\right)-z_{0.25}^{*}\left(u\right),\;\text{where}\;z_{p}^{*}=F^{*-1}\left(u,p\;|\;\left(n\right)\right).$$

From the inferred cdf, *F**(**u**; *z* | (*n*)), it is possible to derive various probability intervals such as the 95% interval [*q*_0.025_; *q*_0.975_], such that,

(A.11) $$\text{Prob}\left\{Z\left(u\right)\in \left[q_{0.025}-q_{0.975}\right]\;|\;\left(n\right)\right\}=0,95$$

with *q*_0.025_ and *q*_0.975_ being the quantiles 0.025 and 0.975, respectively, of cdf. Attribute values referring to the quantiles are estimated from the fit function and of the cutoff values used in the indicator kriging.

Also, uncertainties can be estimated for ranges of attribute values. The probability of a value *Z*(**u**) be within an interval (*a*, *b*] is computed as the difference between the values of cdf for the *b* and *a* thresholds:

(A.12) Prob{Z(u)∈(a,b] ∣ (n)}=F(u,b ∣ (n))−F(u,a ∣ (n)).

After model fittings, indicator kriging procedures were applied to obtain an approximation of the conditional distribution function of the random variables. Based on the estimated function, maps of mollusk spatial distributions along with the corresponding uncertainties for the entire state and also map of estimated prevalence of schistosomiasis were built.

## References

[1] WHO (1985). The Control of Schistosomiasis.

[2] Chitsulo L, Engels D, Montresor A, Savioli L (2000). The global status of schistosomiasis and its control. *Acta Tropica*.

[3] Almeida Machado P (1982). The Brazilian program for schistosomiasis control. *American Journal of Tropical Medicine and Hygiene*.

[4] Klein HS, Pena SDJO (2002). As origens africanas dos escravos brasileiros. *Homo Brasilis: Aspectos Genéticos, Linguísticos, Históricos e Socioantropológicos da Formação do Povo Brasileiro*.

[5] Prado Junior C (1986). *História Econômica do Brasil*.

[6] Rey L (1956). *Contribuição Para o Conhecimento da Morfologia, Biologia e Ecologia dos Planorbídeos Brasileiros Transmissores da Esquistossomose*.

[7] Souza CP, Caldeira RL, Drummond SC (2001). Geographical distribution of *Biomphalaria* snails in the state of minas gerais, Brazil. *Memorias do Instituto Oswaldo Cruz*.

[8] Guimarães RJPS, Freitas CC, Dutra LV (2008). Schistosomiasis risk estimation in Minas Gerais State, Brazil, using environmental data and GIS techniques. *Acta Tropica*.

[9] Tibiriçá SHC, Mitterofhe A, Castro MF (2011). Malacological survey of biomphalaria snails in municipalities along the estrada real in the southeast of the State of Minas Gerais, Brazil. *Revista da Sociedade Brasileira de Medicina Tropical*.

[10] Correa LR, Paraense WL (1971). Susceptibility of *Biomphalaria amazonica* to infection with two strains of *Schistosoma mansoni*. *Revista do Instituto de Medicina Tropical de Sao Paulo*.

[11] Paraense WL, Correa LR (1973). Susceptibility of *Biomphalaria peregrina* from Brazil and Ecuador to two strains of *Schistosoma mansoni*. *Revista do Instituto de Medicina Tropical de Sao Paulo*.

[12] Lutz A (1999). Observações sôbre a evolução do *Schistosoma mansoni*. *Revista Brasileira de Ciências Sociais*.

[13] Katz N, Carvalho OS (1983). Introdução recente da esquistossomose mansoni no sul do estado de Minas Gerais, Brasil. *Memorias do Instituto Oswaldo Cruz*.

[14] Carvalho OS, Rocha RS, Massara CL, Katz N (1988). Primeiros casos autóctones de esquistossomose mansonica em região do noroeste do Estado de Minas Gerais (Brasil). *Saúde Pública da Universidade de São Paulo*.

[15] WHO (1957). *Study Group on the Ecology of Intermediate Snail Hosts of Bilharziasis*.

[16] Paraense WL, Lacaz CS (1972). Fauna planorbídica do Brasil. *Introdução à Geografia Médica do Brasil*.

[17] Rey L (2001). *Parasitologia: Parasitos e Doenças Parasitárias do Homem nas Américas e na África*.

[18] Guimarães RJPS (2010). *Ferramentas de geoprocessamento para o estudo e controle da esquistossomose no Estado de Minas Gerais, biomedicina*.

[19] Guimarães RJPS, Freitas CC, Dutra LV (2006). Analysis and estimative of schistosomiasis prevalence for the state of Minas Gerais, Brazil, using multiple regression with social and environmental spatial data. *Memorias do Instituto Oswaldo Cruz*.

[20] Brooker S (2002). Schistosomes, snails and satellites. *Acta Tropica*.

[21] Beck LR, Rodriguez MH, Dister SW (1997). Assessment of a remote sensing-based model for predicting malaria transmission risk in villages of Chiapas, Mexico. *American Journal of Tropical Medicine and Hygiene*.

[22] Guimarães RJPS, Freitas CC, Dutra LV (2009). Spatial distribution of *Biomphalaria* mollusks at São Francisco River Basin, Minas Gerais, Brazil, using geostatistical procedures. *Acta Tropica*.

[23] Guimarães RJPS, Freitas CC, Dutra LV (2010). A geoprocessing approach for studying and controlling schistosomiasis in the state of Minas Gerais, Brazil. *Memorias do Instituto Oswaldo Cruz*.

[24] Felgueiras CA (1999). *Modelagem ambiental com tratamento de incertezas em sistemas de
informação geográfica: o paradigma geoestatístico por indicação, computação aplicada*.

[25] Camargo ECG (1997). *Desenvolvimento, implementação e teste de procedimentos geoestatísticos (krigeagem) no sistema de processamento de informações georeferenciadas (SPRING) , sensoriamento remoto*.

[26] Camara G, Souza RCM, Freitas UM, Garrido J (1996). Spring: integrating remote sensing and gis by object-oriented data modelling. *Computers and Graphics*.

[27] Souza CP, Lima LC (1990). *Moluscos de Interesse Parasitológico do Brasil*.

[28] Deslandes N (1951). Técnica de dissecção e exame de planorbídeos. *Revista de Saúde Pública*.

[29] Paraense WL, Deslandes N (1955). Observations on the morphology of *Australorbis glabratus*. *Memorias do Instituto Oswaldo Cruz*.

[30] Paraense WL, Deslandes N (1955). Observations on the morphology of *Australorbis nigricans*. *Memorias do Instituto Oswaldo Cruz*.

[31] Paraense WL, Deslandes N (1959). The renal ridge as a reliable character for separating *Taphius glabratus* from *Taphius tenagophilus*. *The American Journal of Tropical Medicine and Hygiene*.

[32] Paraense WL (1975). Estado atual da sistemática dos planorbídeos brasileiros. *Arquivos do Museu Nacional*.

[33] Paraense WL (1981). *Biomphalaria occidentalis* sp. n. from South America (Mollusca Basommatophora Pulmonata). *Memorias do Instituto Oswaldo Cruz*.

[34] Vidigal THDA, Caldeira RL, Simpson AJG, Carvalho OS (2000). Further Studies on the Molecular Systematics of *Biomphalaria* Snails from Brazil. *Memorias do Instituto Oswaldo Cruz*.

[35] Isaaks EH, Srivastava RM (1991). *An Introduction to Applied Geostatistics*.

[36] Deutsch CV, Journel AG (1998). *GSLIB Geostatistical Software Library and User’s Guide*.

[37] Journel AG (1983). Nonparametric estimation of spatial distributions. *Journal of the International Association for Mathematical Geology*.

